# Investigating Metabolic Phenotypes for Sarcoidosis Diagnosis and Exploring Immunometabolic Profiles to Unravel Disease Mechanisms

**DOI:** 10.3390/metabo15010007

**Published:** 2024-12-31

**Authors:** Mohammad Mehdi Banoei, Abdulrazagh Hashemi Shahraki, Kayo Santos, Gregory Holt, Mehdi Mirsaeidi

**Affiliations:** 1 Department of Critical Care Medicine, University of Calgary, Calgary, AB T2N 4Z6, Canada; mmbanoei@ucalgary.ca; 2 Department of Biomedical Engineering, Schulich School of Engineering, University of Calgary, Calgary, AB T2N 1N4, Canada; 3 Division of Pulmonary, Critical Care, and Sleep, College of Medicine—Jacksonville, University of Florida, Jacksonville, FL 32209, USA; hashemishahrak.a@ufl.edu; 4 Division of Pulmonary and Critical Care, University of Miami, Miami, FL 33146, USA; kayohenrique.md@gmail.com (K.S.); gholt@miami.edu (G.H.)

**Keywords:** metabolic phenotype, sarcoidosis, early diagnosis, immunometabolic

## Abstract

**Background:** Sarcoidosis is a granulomatous disease affecting multiple organ systems and poses a diagnostic challenge due to its diverse clinical manifestations and absence of specific diagnostic tests. Currently, blood biomarkers such as ACE, sIL-2R, CD163, CCL18, serum amyloid A, and CRP are employed to aid in the diagnosis and monitoring of sarcoidosis. Metabolomics holds promise for identifying highly sensitive and specific biomarkers. This study aimed to leverage metabolomics for the early diagnosis of sarcoidosis and to identify metabolic phenotypes associated with disease progression. **Methods:** Serum samples from patients with sarcoidosis (*n* = 40, including stage 1 to stage 4), were analyzed for metabolite levels by semi-untargeted liquid chromatography–mass spectrometry (LC-MS). Metabolomics data from patients with sarcoidosis were compared with those from patients with COVID-19 and healthy controls to identify distinguishing metabolic biosignatures. Univariate and multivariate analyses were applied to obtain diagnostic and prognostic metabolic phenotypes. **Results:** Significant changes in metabolic profiles distinguished stage 1 sarcoidosis from healthy controls, with potential biomarkers including azelaic acid, itaconate, and glutarate. Distinct metabolic phenotypes were observed across the stages of sarcoidosis, with stage 2 exhibiting greater heterogeneity compared with stages 1, 3, and 4. **Conclusions:** we explored immunometabolic phenotypes by comparing patients with sarcoidosis with patients with COVID-19 and healthy controls, revealing potential metabolic pathways associated with acute and chronic inflammation across the stages of sarcoidosis.

## 1. Introduction

Sarcoidosis is a multisystem granulomatous disease of unknown etiology, characterized by noncaseating granuloma formations in various organs, especially the lungs. Although the exact cause of sarcoidosis remains unclear, it is thought to result from an exaggerated immune response to an unrecognized antigen. The immune response in sarcoidosis is characterized by the accumulation of T lymphocytes and macrophages, suggesting that pathogenesis primarily revolves around a subset of T cells. While sarcoidosis has systemic involvement, it predominantly manifests in the lungs. The mechanisms underlying immune system activation and response in sarcoidosis remain unknown. Diagnosing sarcoidosis at an early stage is clinically challenging due to its variable presentation and lack of a single reliable diagnostic test [1]. Accurate diagnosis of sarcoidosis is critical, as treatment and prognosis depend on the proper classification. Recently identified biomarkers are now utilized in the diagnosis and monitoring of various diseases [2]. The Scadding system categorizes sarcoidosis into stages according to the chest X-ray findings. Stage 0 shows a normal chest X-ray, while stage 1 displays bilateral hilar lymphadenopathy. Stage 2 presents lymphadenopathy alongside lung granulomas. Stage 3 depicts lung granulomas without lymph node involvement. Stage 4 indicates the progression to pulmonary fibrosis, reflecting advanced lung damage [3]. The classification of sarcoidosis into stages is essential for predicting outcomes and planning treatment strategies, along with monitoring the disease progression over time. Stage 1 is known for its likelihood of resolving on its own without intervention, while stage 4 is associated with poor outcome. The staging process helps healthcare professionals make decisions about treatment options and keeps track of how the disease evolves in each patient while also aiding in educating patients about their condition. However, it is important to note that staging may not always align perfectly with the symptoms experienced by patients or the involvement of organs outside of the lungs. Modern medical practices now utilize imaging techniques and pulmonary function tests to gain a detailed insight into the disease leading to personalized care plans tailored to individual needs and ultimately improving patient outcomes [4]. Blood biomarkers, like metabolites, can significantly help in the staging and management of sarcoidosis by providing valuable insights into disease activity, severity, and prognosis. While no single biomarker is definitive for sarcoidosis, several have shown utility when used in combination.

Several serum biomarkers have been investigated for their diagnostic potential and their role in the pathophysiology of sarcoidosis. Key biomarkers providing significant insights into sarcoidosis and underlying biological mechanisms include serum angiotensin-converting enzyme (SACE), which is notably elevated in sarcoidosis. Although SACE is a sensitive test for sarcoidosis, it lacks specificity. Serum sIL-2R has been studied as a biomarker for sarcoidosis, demonstrating a correlation with disease activity and potential utility in assessing response to treatment [5,6].

Additional serum biomarkers for sarcoidosis include neopterin, lysozyme, human cartilage glycoprotein-39 (YKL40), CD163, CCL18, serum amyloid A [6], and C-reactive protein (CRP) [7]. Neopterin, a marker of cellular immunity activation, is elevated in patients with sarcoidosis and is associated with disease severity. Lysozyme is a macrophage biomarker that increases in sarcoidosis and can serve as an indicator of disease activity [6]. CRP is a systemic inflammation marker associated with sarcoidosis progression and may help identify patients at higher risk for activation [7]. Metabolomics has been proposed as a novel tool to assist in the diagnosis, prognosis, and management of sarcoidosis, and to diagnose disease progression and underlying mechanisms [8,9,10]. Metabolic phenotyping can reflect changes induced by genetic alterations occurring at various stages of sarcoidosis. Metabolomic investigations in sarcoidosis have identified specific metabolic signatures associated with bioenergetic dysregulation, transmethylation, and gut microbiota. Differences in the metabolic profiles of civilian and veteran patients with sarcoidosis further suggest environmental influences on metabolite changes. Metabolomic profiling can aid in differentiating disease stages and monitoring progression, particularly in lung pathologies. Biomarkers such as p-coumaroylagmatine and palmitoylcarnitine have been associated with fibrosing pulmonary involvement in sarcoidosis. NMR-based studies have identified key metabolite alterations, underscoring bioenergetic disruptions and gut microbiota imbalances in patients with sarcoidosis [8,9,10]. Sarcoidosis shares significant immune similarities with COVID-19, particularly in the renin–angiotensin system (RAS) and immune response pathways. In sarcoidosis, elevated ACE levels and granulomatous inflammation align with the pulmonary damage seen in COVID-19, where SARS-CoV-2 uses ACE2 to enter cells. Both diseases involve exaggerated T-helper 1 (Th1) and Th17 immune responses, contributing to inflammation and disease progression. To investigate metabolic alterations related to immunometabolism and inflammatory responses, metabolite changes observed in sarcoidosis were compared with those in COVID-19. This comparison can highlight key insights, as sarcoidosis shares significant immune similarities with COVID-19, particularly in the renin–angiotensin system (RAS) and immune response pathways [11]. We also aimed to determine whether the metabolomic profile could serve as a novel tool for the early diagnosis of sarcoidosis (stage 1) and for identifying specific metabolic phenotypes associated with advanced stages (stages 3 and 4).

## 2. Methods and Materials

### 2.1. Patients’ Enrollment

Serum samples were collected from the patients with active sarcoidosis (*n* = 40) exhibiting clinical signs and symptoms of pulmonary involvement across different stages: stages 1 (*n* = 7), stage 2 (*n* = 13), stage 3 (*n* = 9), and stage 4 (*n* = 11). Sarcoidosis was defined by consistent clinical signs and symptoms of pulmonary involvement, along with a current or past presence of bilateral hilar lymphadenopathy. The diagnosis was further supported by biopsy-confirmed sarcoid-like granulomas in pulmonary samples and the exclusion of other granulomatous conditions, such as mycobacterial infections. Data were collected from patients admitted at the University of Miami Hospital, Miller School of Medicine, Miami, FL, USA.

We compared the sarcoidosis cohort and two control groups: age- and sex-matched patients with COVID-19 (*n* = 40), representing a viral disease with an acute inflammatory response, and a group of 40 healthy individuals. Including these two distinct control cohorts allowed us to evaluate the differences in inflammatory and immune responses between the non-infectious inflammatory condition (sarcoidosis) and COVID-19, and to establish a baseline from healthy controls. Serum samples from virologically confirmed patients with COVID-19 were collected within the first 48 h of hospital admission in 2020 and 2021. Serum samples from patients with sarcoidosis were collected during outpatient visits. Both cohorts were recruited and samples were collected at the University of Miami Hospital, Miller School of Medicine, Miami, FL, USA.

All serum samples were taken in the morning, prior to breakfast, to minimize the impact of recent food or beverage intake on metabolite levels. These protocols were consistently implemented across both patient groups to ensure uniform conditions and enhance the reliability of our metabolomic analysis.

All subjects consented to blood draws under the IRB approval protocol of the University of Miami and the Miami VA IRBs.

### 2.2. Mass Spectrometry Sample Preparation

Regarding serum processing, we followed a strict protocol to ensure sample integrity. After blood was collected, samples were allowed to clot at room temperature for 30 min. Following clotting, the blood was centrifuged at 2000× *g* for 10 min at 4 °C to separate the serum. Serum samples were centrifuged and aliquoted, and immediately stored at −80 °C until further analysis. These procedures were consistently applied to all samples in the study to minimize variability and ensure the reliability of metabolite measurements.

Sample preparation: Step 1: serum samples of 50 μL were transferred to a 96-well plate. Then, 200 μL of 80% ethanol (prepared by mixing 40 μL H_2_O and 160 μL ethanol) was added (Dilution 5) to neutralize any viruses. All plates were centrifuged at 4200 rpm at 4 °C for 10 min to precipitate the proteins and debris. Step 2: 50 μL of extracted supernatant from Step 1 was transferred to each well of a 96-well plate for MS analysis. Then, 150 μL of 40% ethanol (prepared by mixing 90 μL H_2_O and 60 μL ethanol) was added (D4). The plates were sealed and stored at −80 °C until the day of the experiment. The final dilution of samples was D20 (D5 × D4 = D20). Mater mixes (pooled samples) were prepared separately for each cohort, incorporating all samples within the cohort. The MS plate layout was designed to include 3 cohorts per plate, with samples analyzed individually from each cohort. Master mixes were analyzed on each plate to ensure quality control across plates.

### 2.3. Ultra-High Pressure Liquid Chromatography–Mass Spectrometry (UHPLC-MS)-Based Metabolomics Analysis

Serum samples underwent analysis using LC-MS with the Q Exactive HF Hybrid Quadrupole-Orbitrap Mass Spectrometer (Thermo-Fisher, Waltham, MA, USA). Chromatography was conducted utilizing a 2.1 mm × 100 mm long Synchronism HILIC column (Thermo-Fisher) internally packed with 3 µm porous Hyperarc particles.

The elution gradient of acetonitrile with 0.1% formic acid (solvent B) was applied as follows: held at 95% for 2 min, decreased from 85% to 95% over 5 min, then from 5% to 80% over 3 min, held at 5% for 5 min, ramped from 5% to 95% over 2 min, and finally maintained at 95% for the last 3 min. This gradient was applied against 20 mM ammonium formate at pH 3.0 in H_2_O (solvent A). The MS conditions were configured as follows: HESI-II temperature set to 325 °C, auxiliary gas flow at 10 units, sheath gas flow at 25 units, spray voltage of ±2.50 kV, capillary temperature at 275 °C, S-lens RF level at 60%, and auxiliary gas heater temperature at 275 °C for both positive and negative ion modes. Mass scan parameters were set for acquiring mass spectra with a 20 min runtime, utilizing full MS scan type, resolution of 240,000, AGC target set of 3e6, maximum injection time of 200 ms, and a scan range from 70 to 1000 *m*/*z*. All data were acquired in both negative and positive modes. We combined the metabolites for the analysis by removing the overlaps. Metabolomics data acquired by LC-MS were processed using Maven, v12.0 an open-source software tool [12].

### 2.4. Data Analysis

Metabolite identifications and quantification were performed using El-MAVEN V.12 (Elucidata Inc., San Francisco, CA, USA) to measure compound ion intensities [13]. Ion peaks were selected based on the mass-to-charge ratio (*m*/*z*), retention time (RT), and ion intensity of metabolites compared with the pre- and post-blank samples.

For multivariate data analysis, PCA was applied to obtain an overview and find outliers in an unsupervised manner. PLS-DA was used to discriminate phenotypes between the two or more groups. Non-parametric analysis of variance (ANOVA) analysis was performed to detect differences between samples from different groups based on the raw ion intensities, with false discovery rate (FDR) adjustments applied using Bonferroni corrections. Student’s *t*-test was performed to identify differences between samples from the two groups. All statistical tests were two-sided, and adjusted *p*-values below 0.05 were considered statistically significant. The diagnostic values of the metabolites were evaluated by constructing receiver operating characteristic (ROC) curves and computing the areas under the curves (AUCs), alongside sensitivities at predefined specificities. Correlations among variables of interest were performed using the Spearman correlation test, and when necessary, corrected for multiple inferences using Holm’s method. Power analysis was performed to determine the minimum sample size required to detect a statistically significant difference between two populations, based on a user-specified degree of confidence. MetaboAnalyst 6.0 [14], GraphPad Prism 9.5.1, and SIMCA P v 14.0 were used for comprehensive metabolomics data analysis.

## 3. Results

### 3.1. Serum Metabolic Profile for the Diagnosis of Sarcoidosis at Early Stage and Other Stages

**Figure 1:**
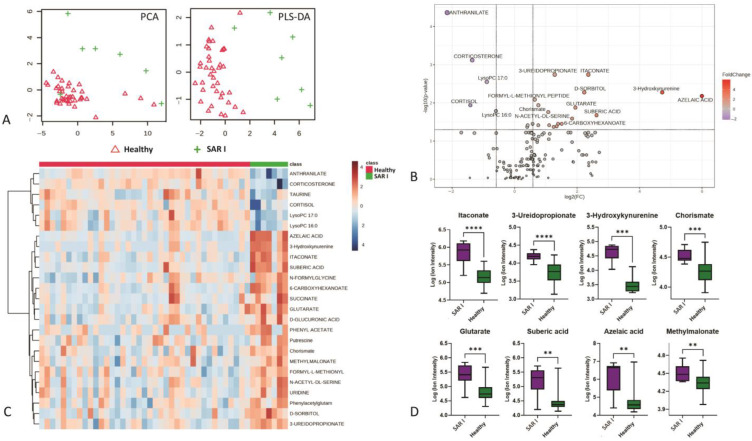
(**A**) PCA and PLS-DA show good predictability to diagnose patients with sarcoidosis at stage 1 compared with healthy control; (**B**) volcano plot shows the significant (*p* < 0.05) metabolites between stage 1 sarcoidosis and normal controls; (**C**) the heatmap shows the difference in top significant metabolites between the two groups; (**D**) the boxplots of some examples of significant metabolites. SAR I—sarcoidosis stage 1. ** *p* < 0.01, *** *p* < 0.001, **** *p* < 0.0001.

**Figure 2:**
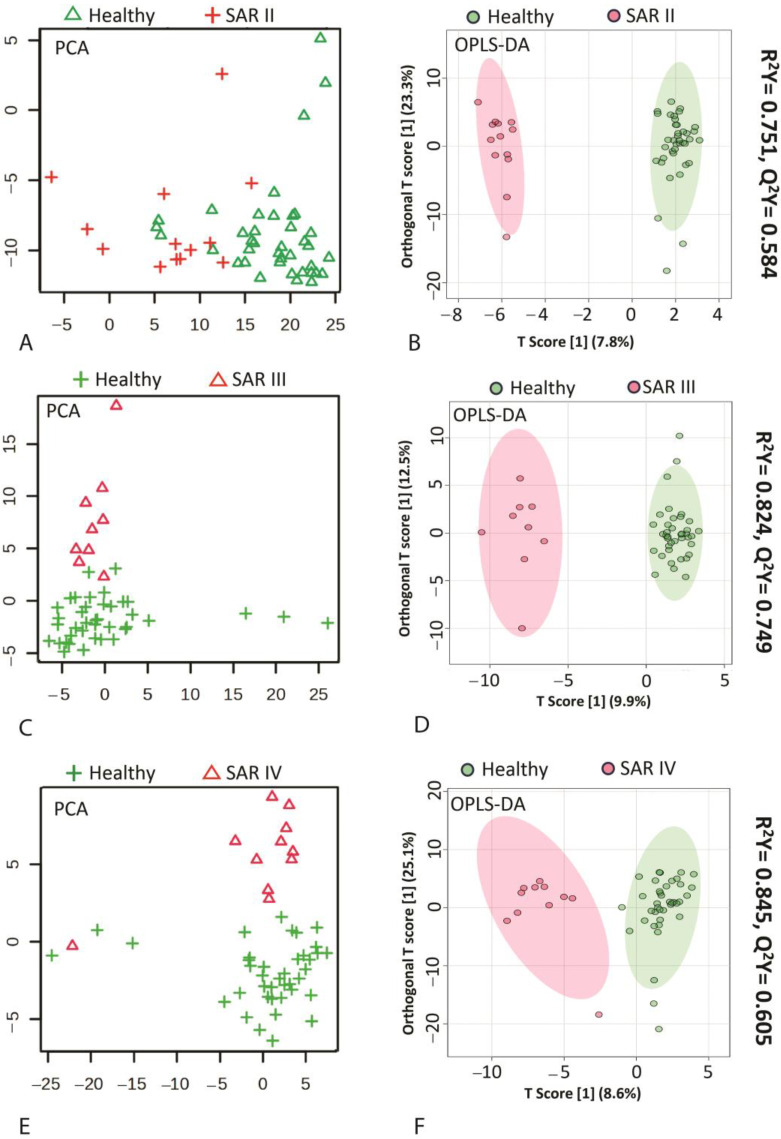
PCA and OPLS-DA show good predictability in diagnosing patients with sarcoidosis at stages 2–4 compared with healthy controls. R^2^Y and Q^2^Y present the goodness of variation and prediction, respectively. Higher Q2 shows a higher predictability for the diagnosis of patients with sarcoidosis at stage 3, indicating a distinct metabolic phenotype compared with patients with sarcoidosis at stages 2 and 4. (**A**,**B**) PCA and OPLS-DA of SAR II vs. healthy. (**C**,**D**) PCA and OPLS-DA of SAR III vs. healthy. (**E**,**F**) PCA and OPLS-DA of SAR IV vs. healthy. SAR II—sarcoidosis stage 2, SAR III—sarcoidosis stage 3, SAR IV—sarcoidosis stage 4.

Our results demonstrate the utility of metabolomics in diagnosing sarcoidosis at an early stage (stage 1). Serum-based metabolomics revealed that patients with stage 1 sarcoidosis can be distinctly diagnosed compared with healthy controls based on the unique metabolic phenotype differentiating the two groups (Figure 1). This includes 55 out of 160 metabolites (*n* = 25, FDR < 0.05) that showed significant changes in patients with stage 1 sarcoidosis compared with controls. Metabolites such as azelaic acid, itaconate, suberic acid, 3-hydroxkynurenic, glutarate, and 3-ureidopropionate emerged as potential biomarkers for diagnosing patients with sarcoidosis at stage 1, each metabolite demonstrating high diagnostic performance individually (ROC > 0.8) (Figure 1D and Table 1). Power analysis confirmed a statistically significant difference in diagnosing stage 1 sarcoidosis compared with healthy controls with the current sample size of stage 1 sarcoidosis (Figure S1). Further analyses revealed significant differences between patients with sarcoidosis with stages 2, 3, and 4 and healthy controls, with stage 3 showing a slightly greater divergence from healthy controls based on the increased number of significant metabolites. Figure 2 illustrates the differences in metabolic phenotypes of stages 2 to 4 compared with the controls using PCA and OPLS-DA (Figure 2A–E). Azelaic acid, suberic acid, 3-hydroxykynurenine, itaconate, and glutarate showed consistent increases among sarcoidosis stages 2 to 4 (Figures S2–S4).

Biomarker analysis was performed to identify the most promising biomarkers for diagnosing patients with sarcoidosis in stages 1 to 4 compared with healthy controls. Table 1 presents six potential metabolite biomarkers for stages 1 to 4, including ROC values for each metabolite, and the cumulative ROC values, sensitivity, and specificity of the combined biomarkers. Further analysis showed that elevated levels of azelaic acid, glutarate, and suberic acid were common across all stages. Increased levels of 3-hydroxykynurenine and itaconate were particularly effective biomarkers in stages 1, 3, and 4 (Figures S5–S8).

### 3.2. Identification of Different Metabolic Phenotypes Related to Sarcoidosis Stages and Immunometabolic Phenotypes

**Figure 3:**
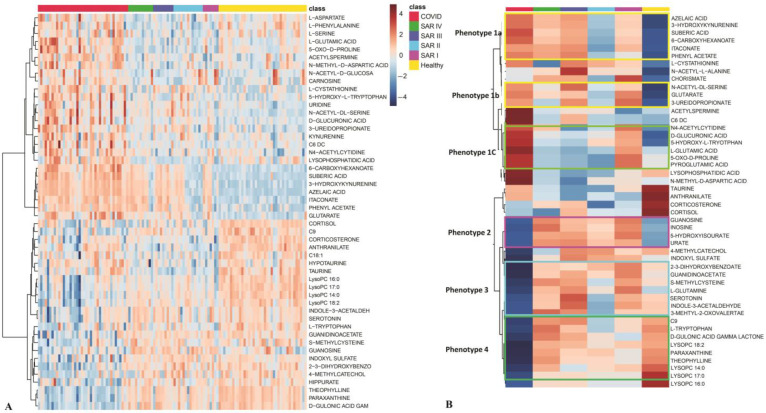
Heatmap analysis based on top 50 most differentiating metabolites. (**A**) All samples of patients with COVID-19, sarcoidosis at 4 different stages (1–4), and healthy controls. (**B**) The average of samples in each group. SAR I—sarcoidosis stage 1, SAR II—sarcoidosis stage 2, SAR III—sarcoidosis stage 3, SAR IV—sarcoidosis stage 4.

Cluster analysis identified four main metabolic phenotypes within the sarcoidosis cohort compared with the patients with COVID-19 and healthy controls, revealing distinct patterns of metabolite alterations (*p* value < 0.05). The clustering highlighted specific metabolic signatures associated with sarcoidosis, contrasting both with the acute inflammatory response observed in COVID-19 and the baseline profiles of healthy controls (Figure 3 and Figure S10, Table S2). Metabolic phenotype 1: This group exhibited elevated concentrations of metabolites in patients with COVID-19, with somewhat elevated levels in patients with sarcoidosis. It included three subgroups (1a, 1b, and 1c), categorized by distinct metabolic patterns across different stages of sarcoidosis (Figure 3B). Phenotype 1a was characterized by elevated metabolite levels in stages 1, 3, and 4 of sarcoidosis. Phenotype 1b showed increased metabolites in stages 1 and 3, while phenotype 1c showed elevated metabolite levels only in stage 1. Phenotype 2 was characterized by increased metabolite levels across all stages of sarcoidosis, with decreases observed in patients with COVID-19 and healthy controls. Phenotype 3 exhibited elevated metabolite levels in stages 1, 3, and 4, along with a slight increase in healthy controls. These metabolites showed a significant decrease in patients with COVID-19 and a slight reduction in sarcoidosis stage 2. Finally, phenotype 4 included metabolites that were elevated in healthy controls and moderately increased in sarcoidosis stages 3 and 4. These metabolites were reduced in sarcoidosis stages 1 and 2 and significantly reduced in patients with COVID-19.

### 3.3. Unraveling of Inflammatory-Related Metabolic Phenotypes of Sarcoidosis Stages

**Figure 4:**
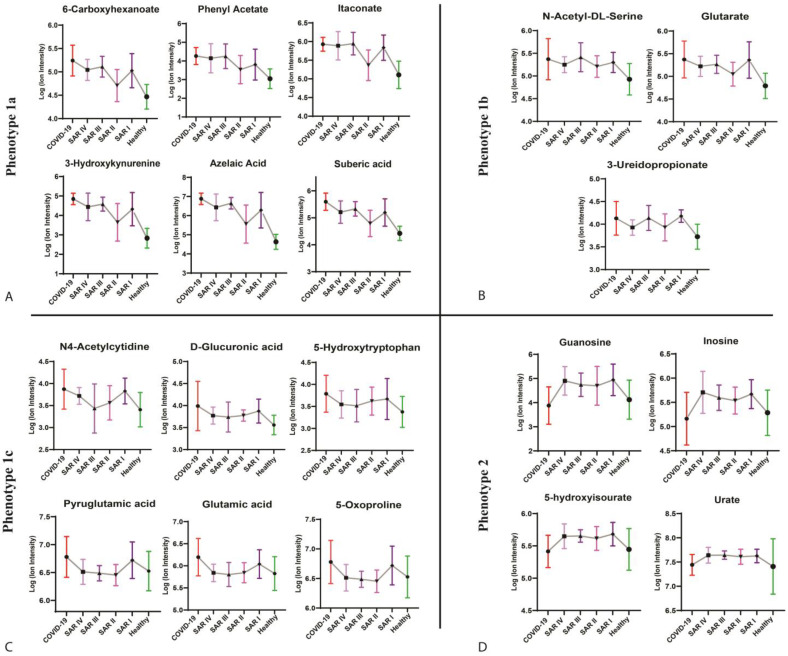
The figure shows the metabolites of each metabolic phenotype 1a (**A**), 1b (**B**), 1c (**C**), and 2 (**D**). Phenotype 1c shows the association of increased metabolites in patients in stages 3 and 4 with a higher level of inflammation. Phenotype 1b shows the association of metabolites in patients in stages 1 and 3 with higher inflammation. Phenotype 1c shows the association of increased metabolites among patients with sarcoidosis in only stage 1 with higher inflammation. Phenotype 2 shows that metabolites are mostly associated with chronic inflammation among all stages of sarcoidosis. SAR I—sarcoidosis stage 1, SAR II—sarcoidosis stage 2, SAR III—sarcoidosis stage 3, SAR IV—sarcoidosis stage 4.

**Figure 5:**
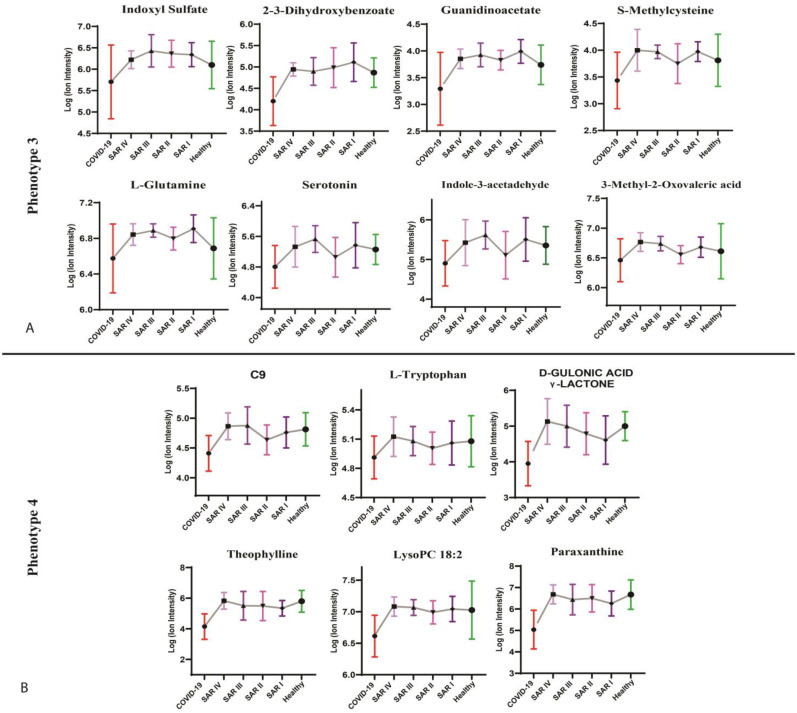
The figure shows the metabolites of each metabolic phenotype 3 (**A**) and 4 (**B**). Metabolic phenotypes 3 and 4 show the association with acute inflammation that are mostly upregulated and downregulated among stages of sarcoidosis, respectively. SAR I—sarcoidosis stage 1, SAR II—sarcoidosis stage 2, SAR III—sarcoidosis stage 3, SAR IV—sarcoidosis stage 4.

Metabolic phenotype 1a may indicate higher concentrations of inflammatory-related metabolites in patients with sarcoidosis in stages 1, 3, and 4 compared with those in stage 2. Our results showed that these metabolites were significantly elevated in COVID-19 and low in health controls, suggesting an association with a more severe disease course, and highlighted inflammatory responses and multi-organ involvement (Figure 4A). The elevated levels of azelaic acid, 3-hydroxykynurenine, suberic acid, 6-carboxyhexanoate, itaconate, and phenyl acetate suggest an upregulation of the dicarboxylic acid and kynurenine pathways, which may be associated with acute phases of inflammation in patients with sarcoidosis at stages 1, 3, and 4 (Figure 4A). Metabolic phenotype 1b highlights shared metabolic pathways between patients with sarcoidosis in stages 1 and 3, which are associated with inflammation (as seen in increased levels in COVID-19) (Figure 4B). These metabolic pathways were not upregulated in patients with sarcoidosis in stages 2 and 4. Metabolites of phenotype 1b that were upregulated in stages 1 and 3 included glutarate, N-acetyl-dl-serine, and 3-ureidopropionate (Figure 4B). Phenotype 1c characterizes patients with sarcoidosis in stage 1, potentially indicating a distinct inflammation-related profile compared with stages 2, 3, and 4. Metabolites associated with this phenotype showed a significant increase in concentration in both patients with COVID-19 and patients with stage 1 sarcoidosis. The upregulation of N4-acetylcysteine, D-glucuronic acid, 5-hydroxy-L-tryptophan, L-glutamic acid, 5-oxo-D-proline, and pyroglutamic acid indicated significant perturbation in L-glutamine metabolism, ascorbate metabolism, and tryptophan metabolism among patients with sarcoidosis in stage 1 (Figure 4C). Figure 5 illustrates the metabolite changes of phenotypes 3 and 4 among patients with sarcoidosis. Metabolic phenotype 3 may be associated with chronic inflammation or specific metabolic pathways, not in all sarcoidosis stages. Most of the metabolites were upregulated, particularly sarcoidosis stages 1, 3, and 4, while they were downregulated in patients with COVID-19 and healthy controls such as L-glutamine, serotonin, and indol-3-acetaldehyde (Figure 5A). Metabolic phenotype 4 may be primarily associated with acute inflammation, as the metabolites were significantly decreased in patients with COVID-19 while remaining elevated in both patients with sarcoidosis and healthy controls. Patients with sarcoidosis in stages 1 and 2 generally showed a decrease in these metabolites compared with the healthy controls such as L-tryptophan, theophylline, and paraxanthine (Figure 5B). The metabolic phenotyping highlighted potential inflammatory-related metabolic profiles across sarcoidosis stages 1 to 4.

### 3.4. Patients with Sarcoidosis in Stage 2 Were a More Heterogeneous and Different Cohort Compared with Patients in Stages 1, 3, and 4

**Figure 6:**
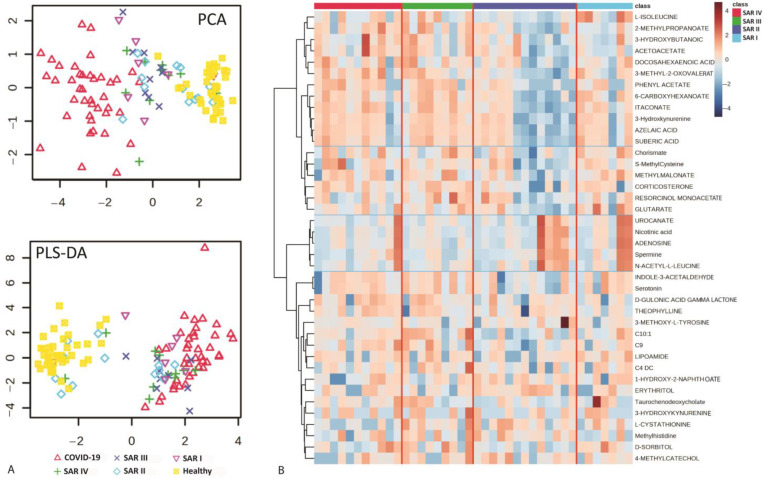
(**A**) PCA and PLS-DA show a different metabolic phenotype of patients with sarcoidosis at stage 2 compared with stages 1, 3, and 4. (**B**) The heatmap shows the heterogeneity of patients with sarcoidosis at stage 2. SAR I—sarcoidosis stage 1, SAR II—sarcoidosis stage 2, SAR III—sarcoidosis stage 3, SAR IV—sarcoidosis stage 4.

Further analysis revealed that patients with sarcoidosis in stage 2 had lower levels of phenotype 1a and 1b metabolites (inflammatory-related metabolites) compared with patients with COVID-19, followed by patients with sarcoidosis in stages 3, 4, and 1. Levels of phenotype 1c metabolites (inflammatory-related metabolites) were elevated in patients with COVID-19 and only in sarcoidosis stage 1. Data from individual patients with sarcoidosis in stage 2 (Figure 6) revealed that stage 2 represented a more heterogeneous cohort compared with stages 1, 3, and 4. This heterogeneity was most pronounced in phenotype 1a. PCA revealed a similar metabolic phenotype between some patients with sarcoidosis in stage 2 and the healthy control (Figure 6A). Additionally, Figure 6 shows that patients with sarcoidosis in stages 3 and 4 exhibited similar metabolic phenotypes.

### 3.5. Metabolic Phenotype 2 Represents the Chronic Inflammation Metabolism Pathways Among All Sarcoidosis Stages

We identified several metabolites that were significantly elevated among patients with sarcoidosis across different stages 1 to 4, while these same metabolites were at lower concentrations and showed no significant change in patients with COVID-19 and healthy controls. Phenotype 2 included metabolites such as guanosine, inosine, 5-hydroxyisourate, and urate, which were primarily elevated in patients with sarcoidosis at stages 1, 3, and 4, with a slight decrease at stage 2 (Figure 4). These data suggest an association between purine metabolism and the inflammatory responses, particularly chronic inflammation (evident by the lack of change in COVID-19), in the pathogenesis of sarcoidosis.

### 3.6. Metabolic Phenotype 3 May Represent the Chronic Inflammation Pathways in Some Sarcoidosis Stages

The metabolic phenotype revealed changes in metabolites that were elevated primarily in patients with sarcoidosis at stages 1 and 3, followed by stage 4, compared with those at stage 2, and healthy controls and patients with COVID-19. This may suggest additional chronic inflammation-related metabolic pathways that are predominantly upregulated in stages 1, 3, and 4. This phenotype showed a decrease in metabolites such as serotonin, indole-3-acetaldehyde, and 3-methyl-2-oxavaleric acid in patients with sarcoidosis at stage 2 and in patients with COVID-19.

### 3.7. Validation of the Current Findings with the Previous Research Study

In the specific context of biomarkers for diagnosing and prognosticating sarcoidosis, validation was conducted through a rigorous comparative analysis between the current data and our previous findings on potential biomarkers used to distinguish this same sarcoidosis patient cohort from COPD [8]. Although the analytical methods differed due to variations in LC-MS parameters and MS instrumentation, strong similarities were observed among the overlapped metabolites in the two studies. The similarities included elevated levels of azelaic acid, glutarate, L-glutamine, uridine, xanthosine, succinate, taurine, anthranilate, and L-cysteine in patients with sarcoidosis, as seen in both the current and previously published studies when compared with healthy controls and patients with COPD. Single biomarker analysis identified N-formylglycine and L-glutamine, each with ROC > 0.8, as potential biomarkers for distinguishing sarcoidosis from COPD. However, a combination of multiple metabolite biomarkers (*n* = 5) achieved a ROC = 0.87, with a sensitivity and specificity of approximately 80% and 79%, respectively (Figure S9).

## 4. Discussion

Metabolomics played a vital role in this study, leading to the identification of metabolic phenotypes in patients with sarcoidosis across stages 1 to 4. For the first time, this approach provided insights into metabolite alterations associated with progression and highlighted potential immune metabolite biomarkers. In this study, metabolomics allowed the early diagnosis of stage 1 sarcoidosis by distinguishing affected individuals from healthy individuals. Our findings suggest that metabolic profiles could be used to predict sarcoidosis progression from stages 2 through 4 compared with the controls. Using a panel of six significant metabolites, we developed robust predictive models for each stage of sarcoidosis. Azelaic acid, glutarate, and suberic acid proved to be particularly critical in distinguishing sarcoidosis stage 1 through stage 4 from healthy individuals. Additionally, 3-hydroxykynurenine and itaconate were elevated in stages 1, 3, and 4. Unique to stage 2 were elevated levels of cystathionine, D-glucuronic acid, and cysteine, highlighting the scope and depth of metabolomic profiling in the diagnosing and understanding of sarcoidosis. Notably, our findings indicate that shared metabolites between sarcoidosis and COVID-19 may be influenced by common metabolic dysregulation, reflecting an immunometabolic phenotype and similar inflammatory cascades. In addition, we identified a unique immunometabolic phenotype across sarcoidosis stages, characterized by elevated metabolites that were not increased in patients with COVID-19 or healthy controls, suggesting an association with chronic inflammation. Further investigations demonstrated that patients with sarcoidosis in stage 2 exhibited greater metabolic heterogeneity compared with patients in stages 1, 3, and 4, suggesting the presence of potential stage-specific biomarkers.

This paper highlights metabolic changes observed in both sarcoidosis and COVID-19, suggesting a biological mechanism associated with pulmonary inflammation. These findings support the idea that these metabolites serve as markers of inflammation, as they are elevated in individuals with sarcoidosis and COVID-19, linking their metabolite profiles to the inflammation processes. Moreover, both diseases exhibit increased activity in pathways associated with the immune response. In COVID-19 and sarcoidosis, the renin–angiotensin system (RAS) and related immune signaling pathways play a vital role in regulating inflammation. The increase in these metabolites correlates with the degree of the inflammatory response, providing insight into the severity. Additionally, the inflammatory microenvironment, characterized by cytokine release and immune cell activation, has been shown to contribute to the metabolic shifts observed in both sarcoidosis and COVID-19, emphasizing the shared inflammatory pathways that connect these two conditions [11].

The most intriguing features revealed by metabolic alterations in stage 1 sarcoidosis compared with healthy controls are variations that offer key insights into disease pathogenesis. These early-stage metabolic changes reveal potential biomarkers in distinguishing stage 1 sarcoidosis from healthy controls, holding promise for early diagnosis and advancing our knowledge of sarcoidosis pathophysiology. Several studies have examined the relationship between metabolomic profiles and disease activity in sarcoidosis. For example, a 2016 study examined whether patients with sarcoidosis exhibit specific metabolic signatures that could impact the granulomatous inflammation characteristic of this disease [10]. This study suggested that dysregulation in bioenergetic processes, transmethylation pathways, and gut microbiota may be associated with sarcoidosis. In addition, recent results indicate a broader connection between the microbiome and respiratory diseases including sarcoidosis, with metabolomic studies revealing potential biomarkers for monitoring the progression of lung tissue-specific pathologies [15]. The metabolomic and metallomic analyses in sarcoidosis have revealed distinct metabolic profile differences between civilian patients and military veterans. These findings suggest that the observed variations between the two cohorts may result from distinct pathophysiological mechanisms, potentially driven by differing environmental exposures faced by civilians and those deployed in military settings. Furthermore, these studies suggest that metabolomic profiling could be a valuable approach for differentiating between stages of radiological sarcoidosis and its progression and peculiarities in this disease among particular populations [8].

Although the precise molecular phenotype for each stage of sarcoidosis has yet to be completely defined, numerous studies are actively investigating potential biomarkers and molecular signatures that reflect the extent of organ involvement and disease activity. This emerging area of research has the potential to enhance our understanding of molecular changes at each stage of sarcoidosis and pave the way for targeted therapeutic approaches and individualized therapy. Among the key metabolites, 3-hydroxykynurenine, a byproduct of tryptophan–kynurenine metabolism, and a known factor in the pathogenesis of neuroinflammation indicates promise for application in sarcoidosis research [16] and systemic inflammation [17]. The dicarboxylic acid pathway, which includes the formation of key metabolites like azelaic acid and suberic acid, is mainly responsible for the biosynthesis of propionic acid (C3:0). These metabolite-related pathways play a major role in regulating inflammation across multiple disease types, including those affecting adipose tissue, neurological systems, and joints, such as osteoarthritis. This highlights the critical role of propionic acid in regulating distinct inflammatory responses associated with various diseases [18,19]. In a study with a relatively large cohort, Rizzi et al. (2024) [20] demonstrated an elevated triglyceride-glucose (TyG) index in patients with sarcoidosis. However, the TyG index was not found to correlate with clinical phenotype, gender, radiological stage, glucocorticoid dose, or treatment regimen. This study concluded that the TyG index can be applied for metabolic assessment in patients with sarcoidosis and for predicting the risk of metabolic syndrome and cardiovascular outcomes [20]. Itaconate, a key immune metabolite, plays the function of modulating the inflammatory response in various diseases, particularly granulomatous conditions such as sarcoidosis [3].

Kass et al. (2018) [21] demonstrated the dysregulation of tryptophan metabolism in sarcoidosis. The reduced levels of tryptophan, along with the upregulation of kynurenine and quinolinate, may reflect the elevated inflammatory state associated with sarcoidosis [21]. This study also revealed reduced levels of serine and threonine metabolisms in sarcoidosis, along with a negative correlation between N-acetylthreonine and FVC, FEV1/FVC, DLCO, and increased Scadding stages [21]. Lower concentrations of methanol and butyrate, along with higher concentrations of lactate, acetate, and N-butyrate discriminated the patient with sarcoidosis from the controls using saliva-based nuclear magnetic resonance (NMR) metabolomics [22]. Using a small sample cohort, NMR analysis revealed that patients with sarcoidosis exhibited a distinct lipid profile compared with the controls [23].

Elevated concentrations of uric acid, the ionized form of urate, have been associated with a wide range of pathological changes and exacerbation of inflammatory processes, including metabolic disorders and chronic inflammatory diseases [24]. Guanosine and inosine are key nucleotides within the purine metabolism pathway and are eventually degraded to form uric acid. Although they have not been well documented for a direct association with chronic inflammation, their role in uric acid production presents an interesting possibility [25].

Although rarely studied in the context of chronic inflammation, 5-hydroxyisourate is of interest due to its role in the uric acid pathway, potentially suggesting its candidacy as a biomarker for chronic inflammatory conditions. Notably, 5-hydroxyisourate is degraded into allantoic acid and ammonia before excretion, indicating its involvement in metabolic pathways associated with inflammation.

Metabolomics examines changes in metabolites within biological samples, including blood, urine, or tissue, providing insights into underlying metabolic pathways and alterations that may contribute to sarcoidosis. Metabolomic technology has been applied across various fields of medicine, including research on the diagnosis and etiology of sarcoidosis [8,9,10,15,26]. Another study applied metabolomics to uncover potential non-invasive biomarkers for the early detection of pulmonary fibrosis in patients with sarcoidosis. Two discriminating metabolites, p-coumaroylagmatine and palmitoylcarnitine, were identified as potential markers for fibrosing pulmonary sarcoidosis [9].

NMR-based metabolomics revealed a significant increase in 3-hydroxybutyrate, acetoacetate, carnitine, cystine, homocysteine, pyruvate, and trimethylamine N-oxide, along with a significant decrease in succinate in patients with sarcoidosis. These findings suggest that these metabolite alterations are associated with disruption in bioenergetic pathways, transmethylation, and gut microbiota in individuals with sarcoidosis [10].

Serum metabolic profiles obtained through NMR spectroscopy effectively differentiated sarcoidosis from tuberculosis (TB). For example, the sera of TB patients had significantly higher levels of lactate, acetate, 3-hydroxybutyrate, glutamate, and succinate, along with significantly lower levels of glucose, citrate, pyruvate, glutamine, and several lipids and membrane metabolites compared with those with sarcoidosis [26].

ACE, serum amyloid A, cytokines, chemokines, and microRNAs have been investigated as potential serum biomarkers in patients with sarcoidosis. While these biomarkers hold promise for aiding in the clinical evaluation of sarcoidosis [27], they currently lack sufficient diagnostic sensitivity and specificity.

The inflammatory phenotypes of sarcoidosis stages are complex and multifaceted, involving a diverse array of immune responses and disease characteristics [28]. Metabolic phenotypes and potential biomarkers of sarcoidosis show promise for use in the differential diagnosis of sarcoidosis among several conditions such as infectious granulomatous lung disease [29], hypersensitivity pneumonitis, autoimmune diseases, and malignancy [30] that can present with similar clinical, radiological, and pathological findings.

The metabolites identified in this study, including S-methylcysteine, 2-3-dihydrobenzoate, suberic acid, and xanthine, have been classified as xenobiotics within human metabolic processes. These metabolites, which can be influenced by factors such as diet and medications, may play a role in human biological functions, especially when in disease conditions. While our study cohorts included data on medication histories but not dietary habits, this study focused on analyzing endogenous metabolites and xenobiotic exposure differences between patients with sarcoidosis, COVID-19, and healthy controls. However, additional analyses and study is required to evaluate the influence of medications and dietary sources on observed metabolic profiles. Nonetheless, there is evidence suggesting that certain xenobiotics, such as those mentioned above, play a role in biological and pathophysiological processes. For instance, S-methylcysteine (SMC) has biological relevance in humans, particularly in detoxification processes. SMC is a precursor to important metabolites and possesses antioxidant properties under oxidative stress conditions by enhancing the activity of detoxification enzymes such as glutathione peroxide and catalase [31]. 2-3-dihydrobenzoate (2,3-DHBA) is a metabolite of aspirin metabolism associated with inflammatory responses and various metabolic pathways. It can exhibit anti-inflammatory activity under inflammatory conditions like sarcoidosis [32]. Suberic acid, although primarily known as a plasticizer and present only in trace amounts in human tissue, has been shown to play a potential role in fatty acid metabolism. It has demonstrated anti-inflammatory effects by modulating key signaling pathways, such as NF-kB and MAPK, and promoting collagen synthesis in UNB-irritated cells. It also influences fatty acid metabolism and mitochondrial function associated with inflammatory response. Elevated levels of suberic acid could indicate metabolic imbalances and disruption in fatty acid oxidation, which are associated with obesity and insulin resistance [33]. Xanthine, like theophylline, has dietary sources such as coffee and tea. However, elevated levels of xanthine may reflect both the dietary and the therapeutic use of theophylline for managing inflammation. Xanthine and xanthine oxidase play crucial roles in promoting and sustaining inflammatory responses in humans through multiple mechanisms including ROS generation and uric acid production [34].

Limitations of this study include small sample size (especially for different stages of sarcoidosis), the lack of age- and sex-matched patients with varying stages of sarcoidosis, the absence of a validation study using a larger cohort, and the need to quantify a broader range of metabolites, particularly lipids. Additionally, comprehensive cytokine profiling of the study cohorts could enhance the understanding of the complex relationship between metabolites and inflammatory responses.

## 5. Conclusions

This study provides evidence supporting the role of metabolomics analysis in elucidating the pathophysiology of sarcoidosis and identifying potential metabolite biomarkers associated with acute and chronic inflammatory responses. By advancing our understanding of these metabolic pathways and their associations with inflammation, we move closer to unlocking novel diagnostic and therapeutic avenues that can improve patient outcomes in sarcoidosis and beyond.

## Figures and Tables

**Table 1:** The most potential metabolites with high ROC values using single (each metabolite) and multi (all 6 metabolites). The numbers in the table present the average of AUC values and the numbers in parentheses present the minimum and maximum values of the obtained AUC of each metabolite. According to the high AUC values (>0.9), all metabolites have high sensitivity to distinguish the sarcoidosis stages.

Metabolites	Stage 1	Stage 2	Stage 3	Stage 4
Azelaic acid	0.897 (0.665–1)	0.79 (0.612–0.937)	1.0 (1–1)	0.972 (0.902–1)
3-Hydroxykynurenine	0.903 (0.692–1)		0.991 (0.957–1)	0.939 (0.0808–1)
Glutarate	0.879 (0.639–1)	0.771 (0.609–0.893)	0.915 (0.818–0.915)	0.897 (0.795–0.966)
Suberic acid	0.857 (0.582–1)	0.731 (0.524–0.894)	0.997 (0.923–1)	0.928 (0.79–1)
3-Ureidopropionate	0.938 (0.832–0.99)			
Itaconate	0.941 (0.802–1)		0.97 (0.889–1)	0.932 (0.803–1)
L-Cystathionine		0.796 (0.627–0.929)		
D-Glucuronic acid		0.805 (0.633–0.916)		
6-Carboxyhexanoate			0.969 (0.913–1)	0.944 (0.864–1)
L-Cysteine		0.783 (0.641–0.887)		
All metabolites	0.921 (0.621–1)	0.93 (0.71–1)	0.989 (0.932- 1)	0.951 (0.808–1)
Sensitivity	86	91	100	100
Specificity	92	97	100	100

## Data Availability

The data presented in this study are available on request from the corresponding author. The data are not publicly available due to privacy.

## Supplementary Materials

The following supporting information can be downloaded at: https://www.mdpi.com/article/10.3390/metabo15010007/s1. Figure S1: Power analysis using the most significant metabolites shows high predicted power (>0.8) with a sample size estimation of 7 samples; Figure S2: The heatmap shows the top 40 significant metabolites between sarcoidosis patients in stage 2 versus healthy control; Figure S3: The heatmap shows the top 40 significant metabolites between sarcoidosis patients in stage 3 versus healthy control; Figure S4: The heatmap shows the top 40 significant metabolites between sarcoidosis patients in stage 2 versus healthy control; Figure S5: A: Top single metabolite biomarkers to identify sarcoidosis stage 1 from healthy controls, B: ROC based on the 6 metabolites C: The predicted class probabilities (average of the cross-validation) for each sample using the best classifier (based on AUC). The confusion matrix is also provided below the classifier image; Figure S6: A: Top single metabolite biomarkers to identify sarcoidosis stage 2 from healthy controls, B: ROC based on the 6 metabolites C: The predicted class probabilities (average of the cross-validation) for each sample using the best classifier (based on AUC). The confusion matrix is also provided below the classifier image; Figure S7: A: Top single metabolite biomarkers to identify sarcoidosis stage 3 from healthy controls, B: ROC based on the 6 metabolites C: The predicted class probabilities (average of the cross-validation) for each sample using the best classifier (based on AUC). The confusion matrix is also provided below the classifier image; Figure S8: A: Top single metabolite biomarkers to identify sarcoidosis stage 4 from healthy controls, B: ROC based on the 6 metabolites C: The predicted class probabilities (average of the cross-validation) for each sample using the best classifier (based on AUC). The confusion matrix is also provided below the classifier image; Figure S9: A: ROC based on the 6 metabolites to identify Sarcoidosis from COPD, B: The predicted class probabilities (average of the cross-validation) for each sample using the best classifier (based on AUC). The confusion matrix is also provided below the classifier image. C: ROC based on the different combinations of different metabolites. D: Significant metabolites and their importance in identifying Sarcoidosis from COPD; Figure S10: Schematic of the four primary metabolic phenotypes highlighting the distinct metabolic profiles across different stages of sarcoidosis, COVID-19, and healthy controls; Table S1: Unpaired *t*-Test analysis between sarcoidosis patients in stage 1 and healthy control; Table S2: The 4 main phenotypes are possibly linked to immune activation. Each phenotype is characterized by changes in among COVID-19, 4 stages of sarcoidosis and healthy controls through symbols (=): equal concentrations, (>), Increased concentrations and (<): decreased concentrations across the different groups.
