# Supplementary material for: Investigating Metabolic Phenotypes for Sarcoidosis Diagnosis and Exploring Immunometabolic Profiles to Unravel Disease Mechanisms
Source: Metabolites. 2024 Dec 31;15(1):7. doi: 10.3390/metabo15010007 (PMC11766916; doi:10.3390/metabo15010007)
Supplement: Supplementary file 1 [file metabolites-15-00007-s001.zip › metabolites-3260350-supplementary.pdf]

metabolites

MDPI

Table S1. Unpaired *t*-Test analysis between sarcoidosis patients in stage 1 and healthy control.

	Name	Mean (SD) of Healthy	Mean (SD) of One	*p*-value	Fold Change	Healthy/One
1	FORMYL-L-METHIONYL PEPTIDE	19102.936 (6564.893)	29861.350 (5923.065)	0.0002	−1.56	Down
2	LysoPC 16:0	51832777.897 (13019426.270)	34257058.286 (9619002.714)	0.0015	1.51	Up
3	NALPHA-ACETYL-L-LYSINE	42985.413 (12054.959)	56641.780 (14662.220)	0.0105	−1.32	Down
4	ITACONATE	164166.886 (91886.122)	841857.631 (480392.369)	< 0.0001 (W)	−5.13	Down
5	3-UREIDOPROPIONATE	6461.595 (3633.278)	15740.541 (4831.959)	< 0.0001 (W)	−2.44	Down
6	ANTHRANILATE	33231.772 (31452.474)	7442.465 (4416.900)	< 0.0001 (W)	4.47	Up
7	CORTICOSTERONE	16668.515 (6219.837)	6509.030 (3096.996)	< 0.0001 (W)	2.56	Up
8	LysoPC 17:0	708285.564 (264255.267)	382246.666 (112029.207)	< 0.0001 (W)	1.85	Up
9	D-SORBITOL	215704.509 (257024.209)	1011675.940 (1205170.096)	0.0002 (W)	−4.69	Down
10	3-Hydroxkynurenine	1629.496 (2893.591)	43093.207 (29597.919)	0.0002 (W)	−26.45	Down
11	AZELAIC ACID	71282.373 (94544.748)	4543821.061 (3458426.989)	0.0003 (W)	−63.74	Down
12	Chorismate	20372.081 (10076.519)	34490.951 (9689.586)	0.0007 (W)	−1.69	Down
13	CORTISOL	32069.916 (15210.377)	12010.359 (9204.340)	0.0008 (W)	2.67	Up
14	GLUTARATE	80031.659 (75165.828)	308365.203 (228269.940)	0.0010 (W)	−3.85	Down
15	N-ACETYL-DL-SERINE	105608.926 (57664.466)	222865.623 (116279.337)	0.0015 (W)	−2.11	Down
16	SUBERIC ACID	37632.131 (66723.865)	231655.107 (169633.779)	0.0019 (W)	−6.16	Down
17	6-CARBOXYHEXANOATE	36875.212 (33010.973)	132161.239 (71979.778)	0.0025 (W)	−3.58	Down
18	N-ALPHA-ACETYL-L-ASPARAGINE	3886.143 (4055.885)	11057.086 (7054.326)	0.0039 (W)	−2.85	Down
19	L-CYSTEINE	1522.649 (1411.146)	3993.527 (3514.880)	0.0040 (W)	−2.62	Down
20	METHYLMALONATE	22581.959 (7646.284)	33662.303 (11954.453)	0.0041 (W)	−1.49	Down
21	N-FORMYLGLYCINE	14111.010 (5473.470)	23854.210 (9239.941)	0.0051 (W)	−1.69	Down
22	ALPHA-D-GLUCOSE	32890808.103 (10784865.609)	43981478.000 (10442413.256)	0.0051 (W)	−1.34	Down
23	D-GLUCURONIC ACID	4171.973 (2126.886)	8982.779 (6292.622)	0.0051 (W)	−2.15	Down
24	Phenylacetylglutamine	470339.399 (357226.514)	1197109.553 (701611.165)	0.0057 (W)	−2.55	Down
25	2-OXOBUTANOATE	2067158.167 (655470.935)	2730385.714 (665399.649)	0.0064 (W)	−1.32	Down
26	N4-Acetylcytidine	3448.883 (2252.960)	8192.419 (5359.620)	0.0065 (W)	−2.38	Down
27	N-ACETYL-L-ALANINE	295483.694 (141145.114)	431185.257 (120013.132)	0.0098 (W)	−1.46	Down
28	SUCCINATE	1129566.525 (2031932.306)	3915035.549 (4651370.621)	0.0109 (W)	−3.47	Down

29	ETHYLMALONIC ACID	584289.196 (192480.447)	802843.124 (246972.099)	0.0109 (W)	−1.37	Down
30	THEOPHYLLINE	1116008.487 (833839.373)	341753.849 (276981.190)	0.0109 (W)	3.27	Up
31	L-ANSERINE	1460.071 (3837.217)	4803.169 (7196.327)	0.0118 (W)	−3.29	Down
32	4-ACETAMIDOBUTA-NOATE	37632.788 (14371.858)	54307.417 (16765.081)	0.0120 (W)	−1.44	Down
33	ALLOSE	28091141.846 (8680803.158)	36096538.857 (7438574.004)	0.0120 (W)	−1.28	Down
34	N-ACETYL-D-GLU-COSAMINE	32434.578 (112338.594)	166437.757 (289836.553)	0.0120 (W)	−5.13	Down
35	PHENYL ACETATE	2700.181 (5025.452)	16122.972 (16256.239)	0.0123 (W)	−5.97	Down
36	L-GLUTAMINE	5848479.295 (2424402.949)	8495619.929 (2725828.652)	0.0133 (W)	−1.45	Down
37	TAURINE	3163523.115 (1171525.531)	2104623.213 (866597.496)	0.0146 (W)	1.5	Up
38	XANTHOSINE	48366.757 (168095.127)	230970.471 (406125.646)	0.0146 (W)	−4.78	Down
39	Putrescine	41250.330 (23156.067)	78931.971 (41097.113)	0.0146 (W)	−1.91	Down
40	GUANOSINE	82883.094 (203739.565)	180556.400 (200025.104)	0.0157 (W)	−2.18	Down
41	4-HYDROXY-L-PRO-LINE	41095.758 (23362.889)	62789.107 (17304.489)	0.0176 (W)	−1.53	Down
42	TRANS-4-HYDROXY-PROLINE	41095.758 (23362.889)	62789.107 (17304.489)	0.0176 (W)	−1.53	Down
43	C4 DC	19857.833 (13350.941)	36646.449 (17175.384)	0.0176 (W)	−1.85	Down
44	N-ACETYL-L-ASPARTIC ACID	580687.755 (2192500.018)	3220474.604 (5819386.044)	0.0194 (W)	−5.55	Down
45	N-ACETYL-D-GALAC-TOSAMINE	46098.542 (164831.570)	226407.813 (410123.310)	0.0194 (W)	−4.91	Down
46	INOSINE	329499.698 (368199.490)	561904.964 (370732.785)	0.0212 (W)	−1.71	Down
47	CYTIDINE	8327.275 (31545.231)	42355.274 (73252.855)	0.0218 (W)	−5.09	Down
48	5-hydroxyisourate	348849.026 (212631.265)	518142.067 (220623.516)	0.0253 (W)	−1.49	Down
49	C-glycosyltryptophan	915.026 (615.967)	1953.960 (1225.078)	0.0266 (W)	−2.14	Down
50	TRANS-ACONITATE	71224.938 (43369.385)	112820.117 (46596.616)	0.0276 (W)	−1.58	Down
51	Aconitic	71224.938 (43369.385)	112820.117 (46596.616)	0.0276 (W)	−1.58	Down
52	URIDINE	532330.566 (186246.224)	880895.136 (405546.711)	0.0301 (W)	−1.65	Down
53	CARNOSINE	71517.531 (301306.432)	256078.439 (521997.904)	0.0354 (W)	−3.58	Down
54	L-ARGININE	1467508.345 (769176.534)	2420702.321 (1455419.594)	0.0355 (W)	−1.65	Down
55	2-METHYLMALEATE	25262.483 (13054.921)	35761.737 (12873.802)	0.0488 (W)	−1.42	Down

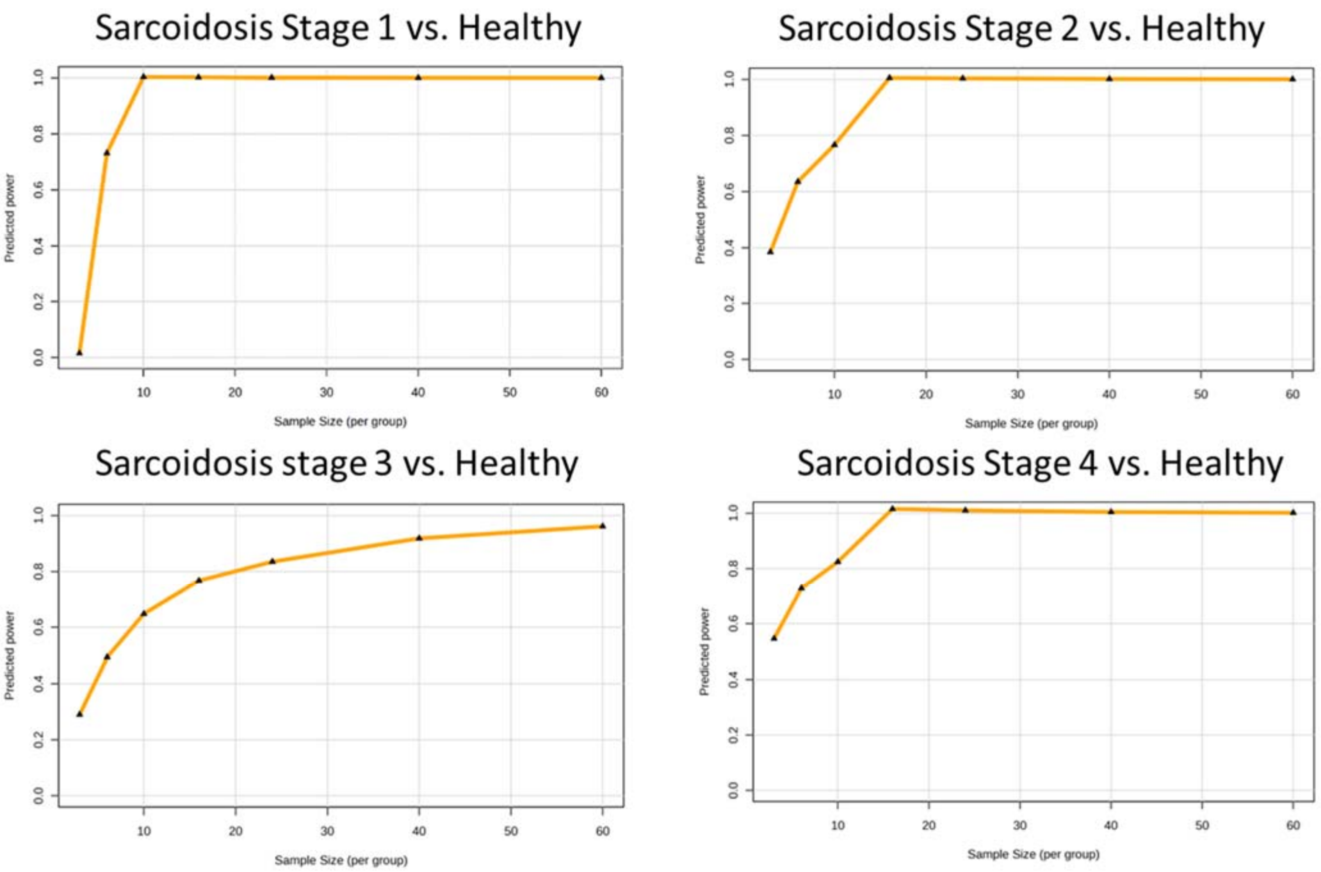

Figure S1. Power analysis using the most significant metabolites shows high predicted power (> 0.8) with sample size estimation of 7 samples.

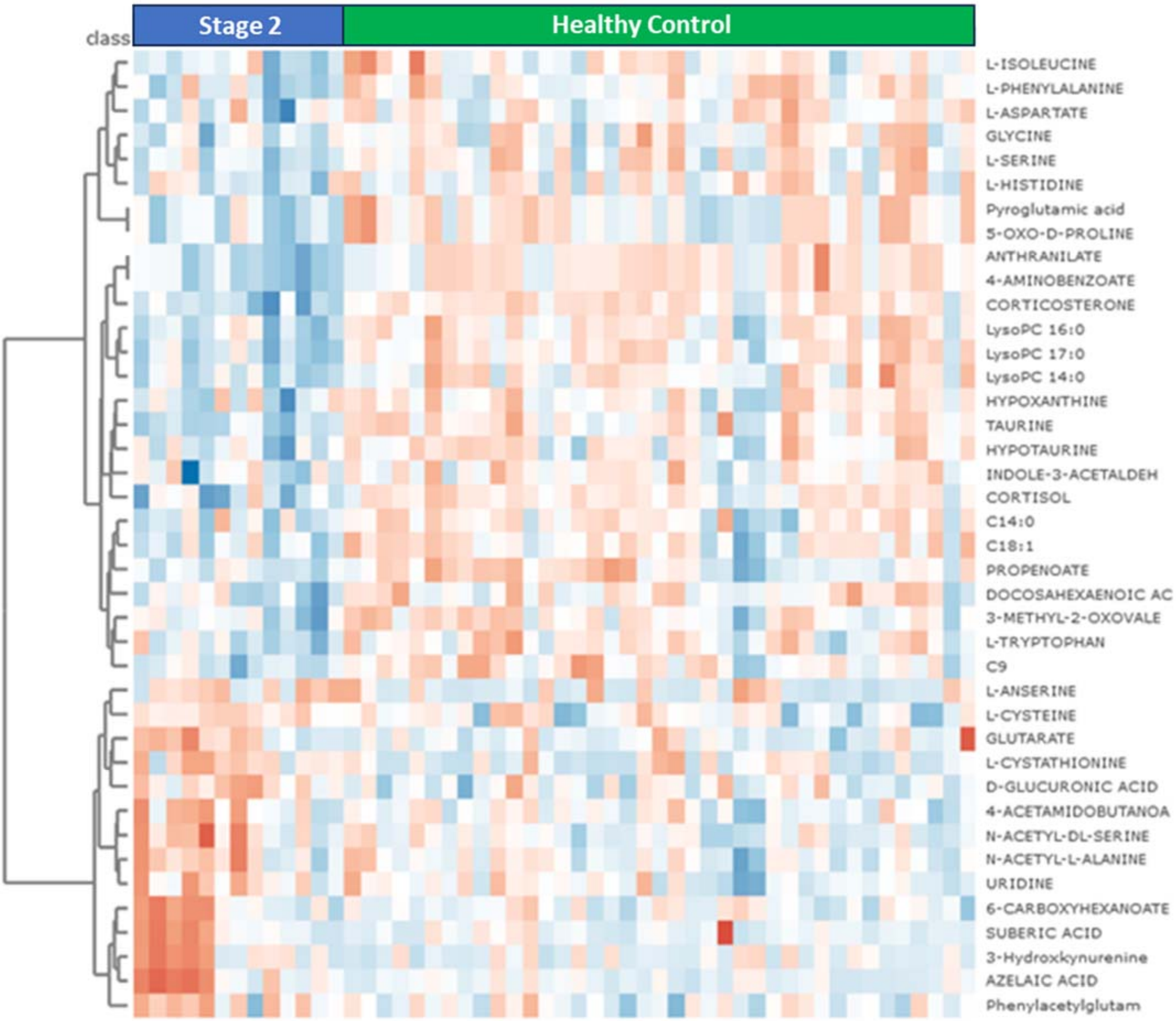

Figure S2. The heatmap shows the top 40 significant metabolites between sarcoidosis patients in stage 2 versus healthy control.

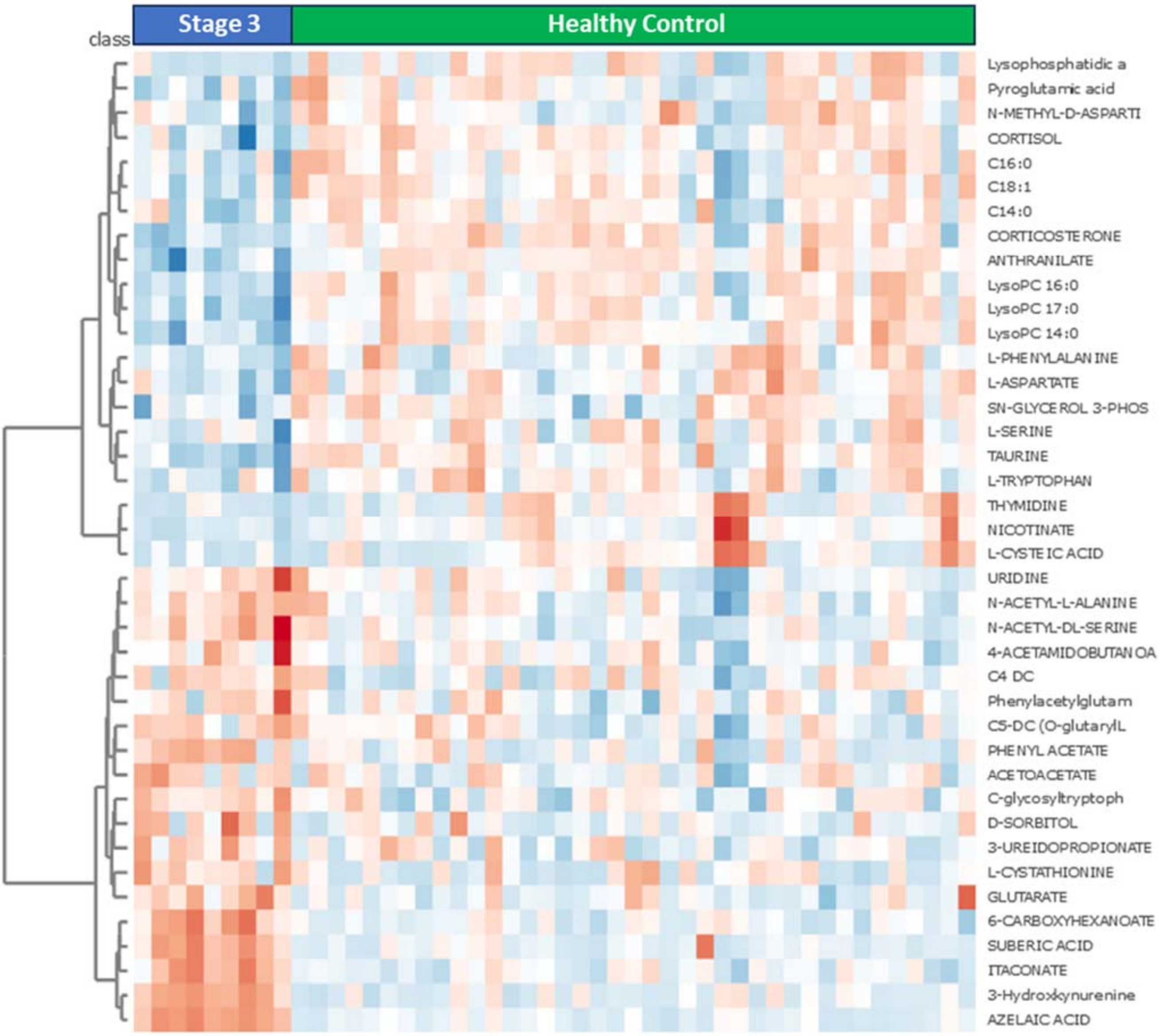

Figure S3. The heatmap shows the top 40 significant metabolites between sarcoidosis patients in stage 3 versus healthy control.

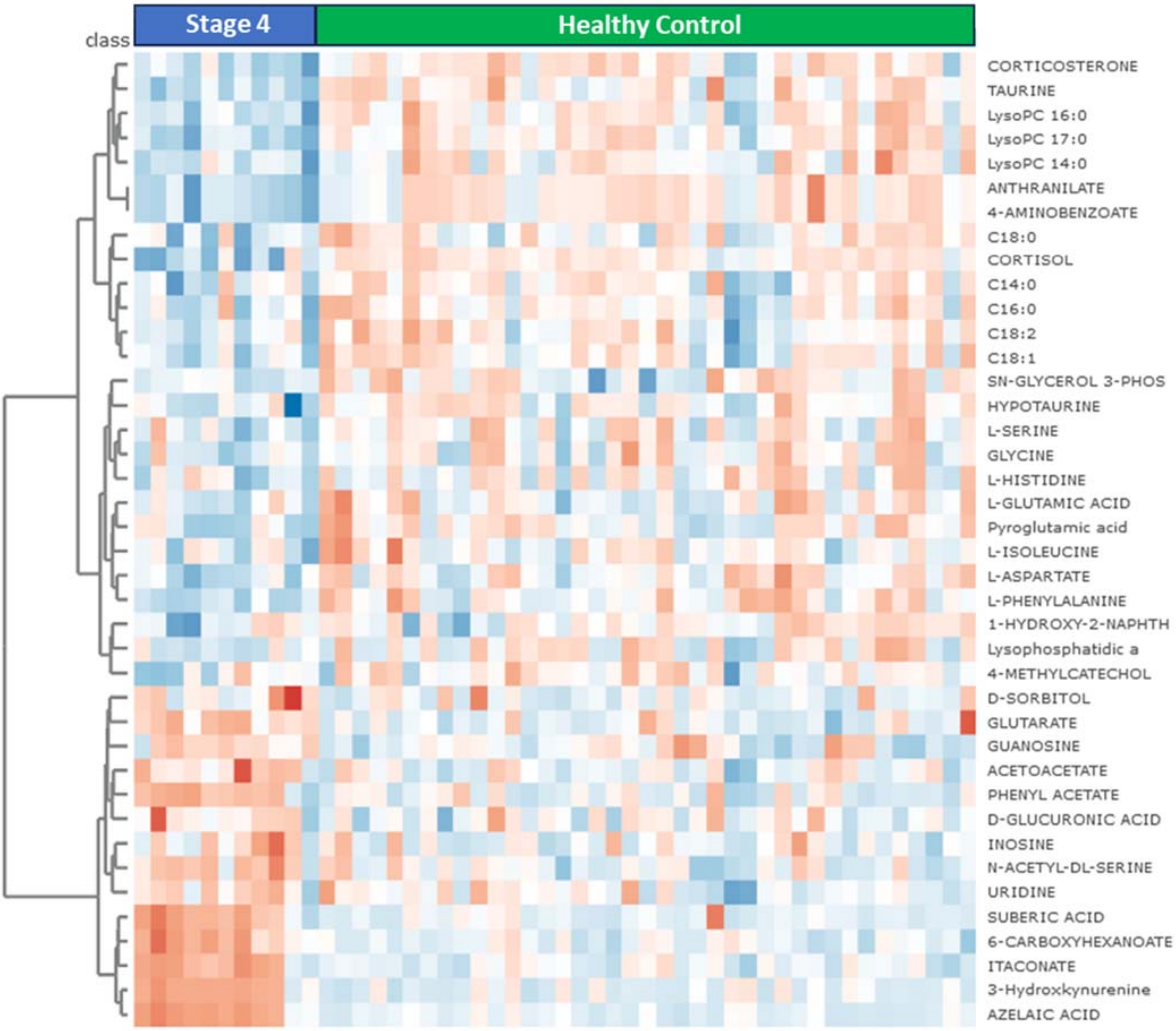

Figure S4. The heatmap shows the top 40 significant metabolites between sarcoidosis patients in stage 2 versus healthy control.

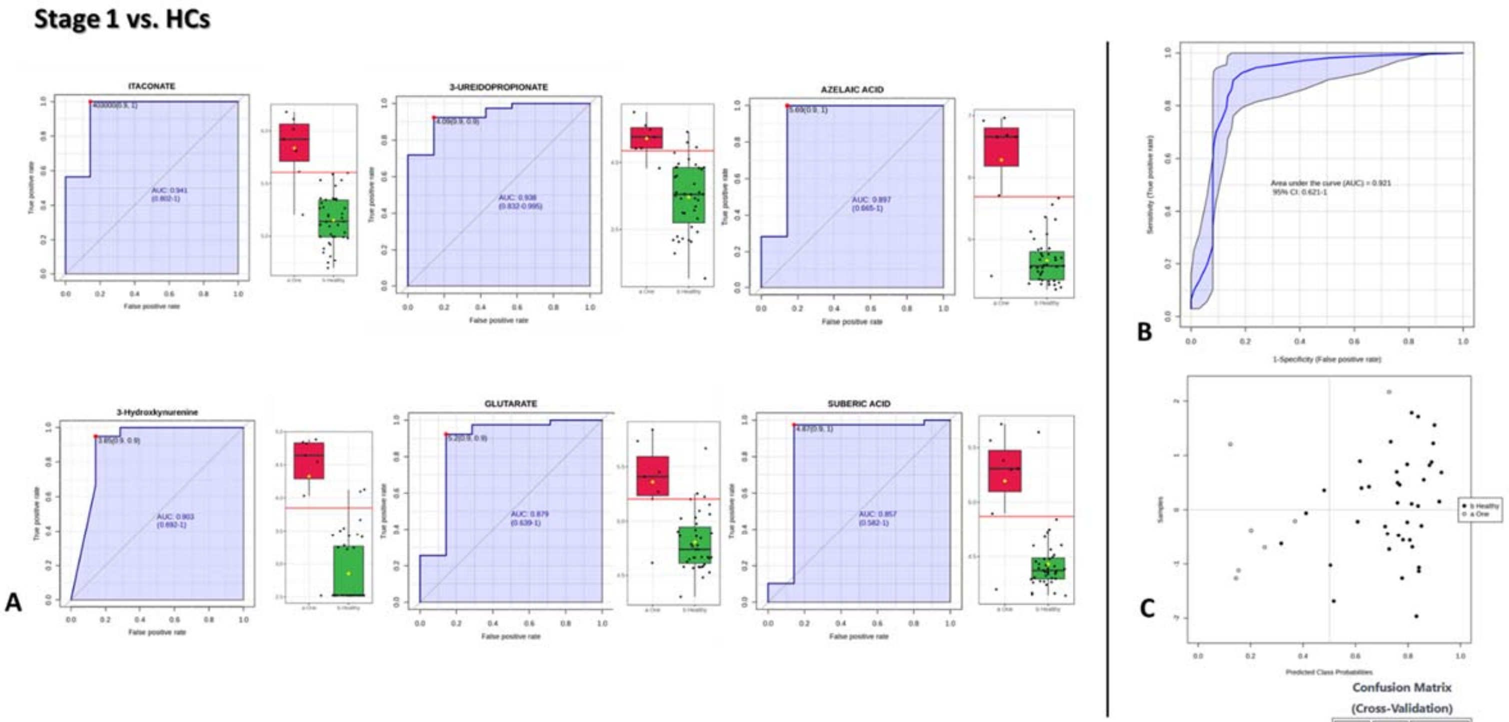

Confusion Matrix
(Cross-Validation)

	0	1
0	6	3
1	1	36

Figure S5. A: Top single metabolite biomarkers to identify sarcoidosis stage 1 from healthy controls, B: ROC based on the 6 metabolites C: The predicted class probabilities (average of the cross-validation) for each sample using the best classifier (based on AUC). The confusion matrix is also provided below the classifier image.

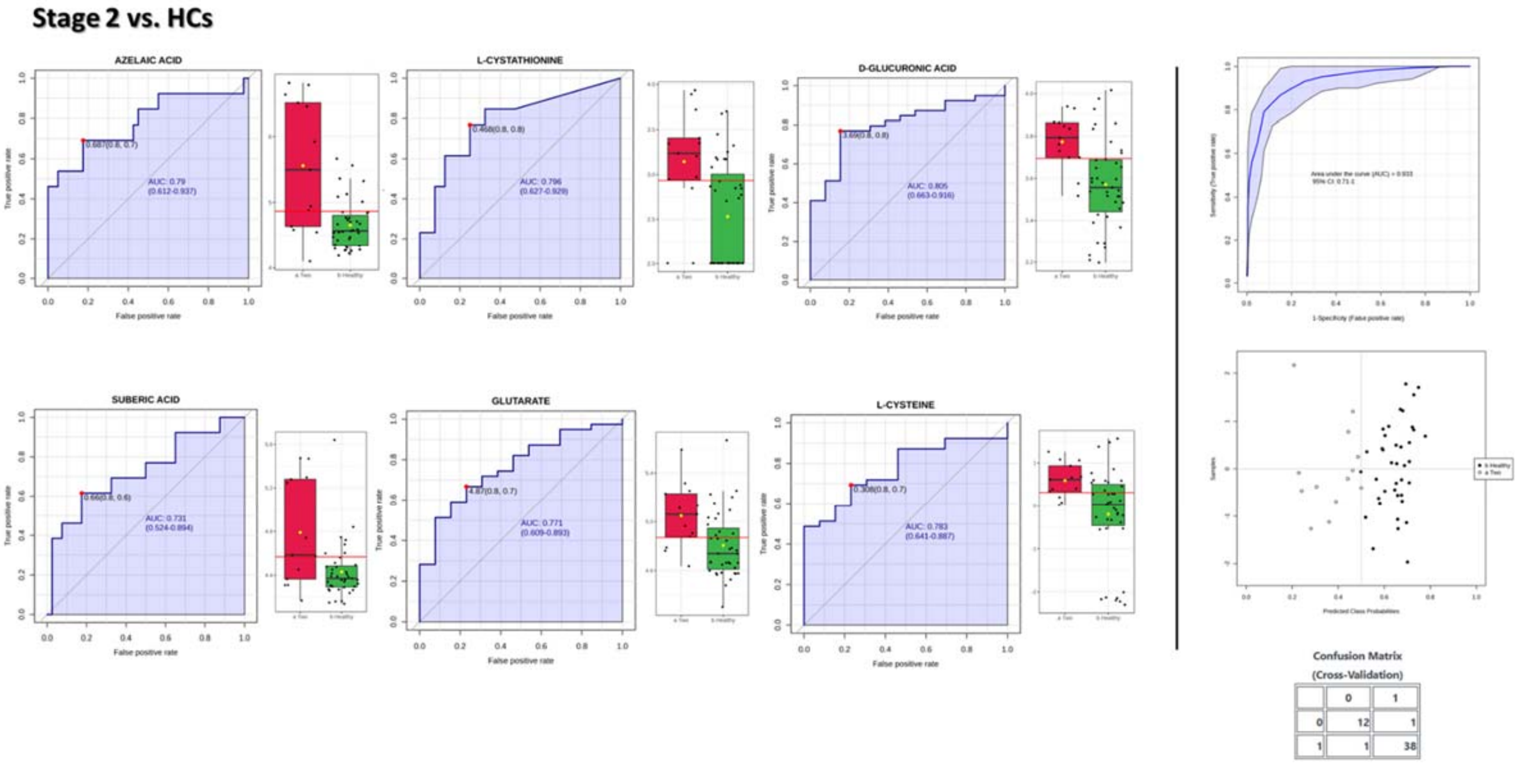

Figure S6. A: Top single metabolite biomarkers to identify sarcoidosis stage 2 from healthy controls, B: ROC based on the 6 metabolites C: The predicted class probabilities (average of the cross-validation) for each sample using the best classifier (based on AUC). The confusion matrix is also provided below the classifier image.

Stage 3 vs. HCs

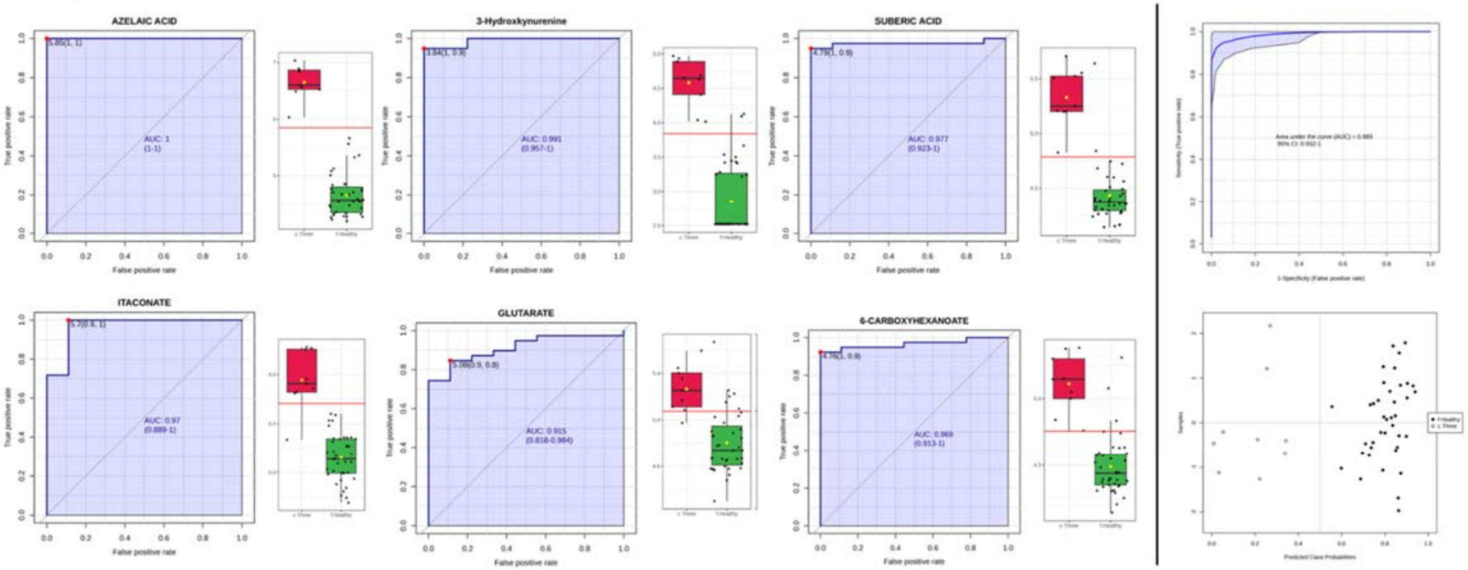

Confusion Matrix
(Cross-Validation)

	0	1
0	9	0
1	0	39

Figure S7. A: Top single metabolite biomarkers to identify sarcoidosis stage 3 from healthy controls, B: ROC based on the 6 metabolites C: The predicted class probabilities (average of the cross-validation) for each sample using the best classifier (based on AUC). The confusion matrix is also provided below the classifier image.

Stage 4 vs. HCs

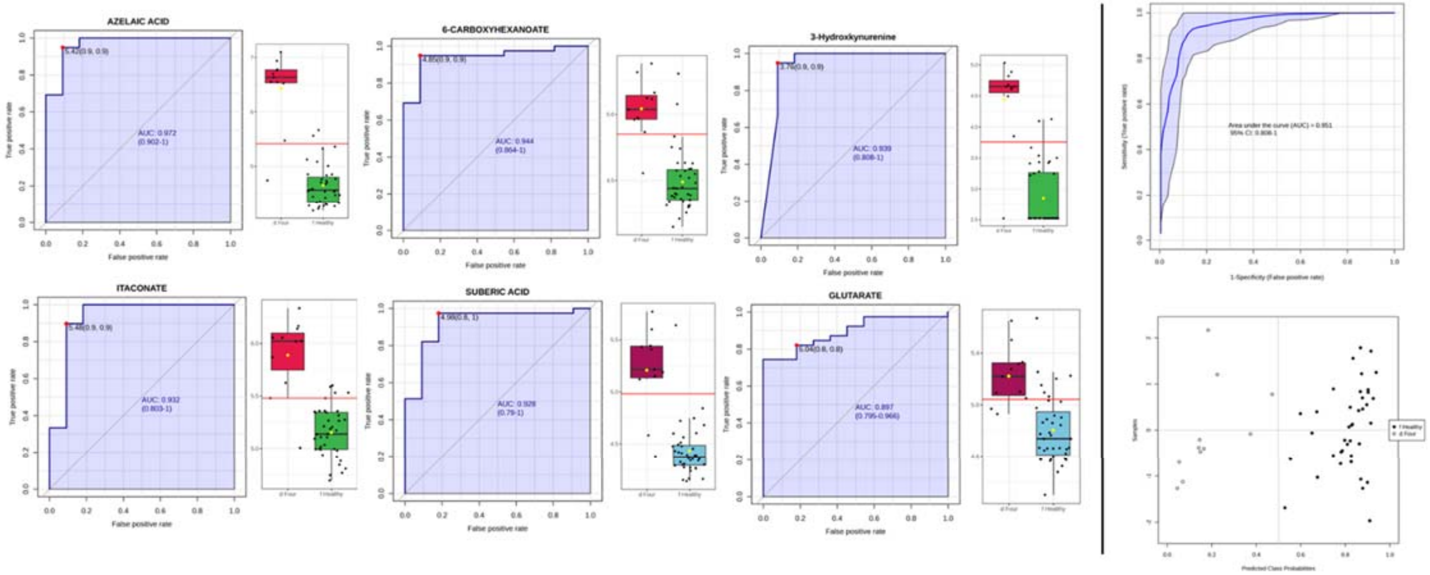

Confusion Matrix
(Cross-Validation)

	0	1
0	11	0
1	0	39

Figure S8. A: Top single metabolite biomarkers to identify sarcoidosis stage 4 from healthy controls, B: ROC based on the 6 metabolites C: The predicted class probabilities (average of the cross-validation) for each sample using the best classifier (based on AUC). The confusion matrix is also provided below the classifier image.

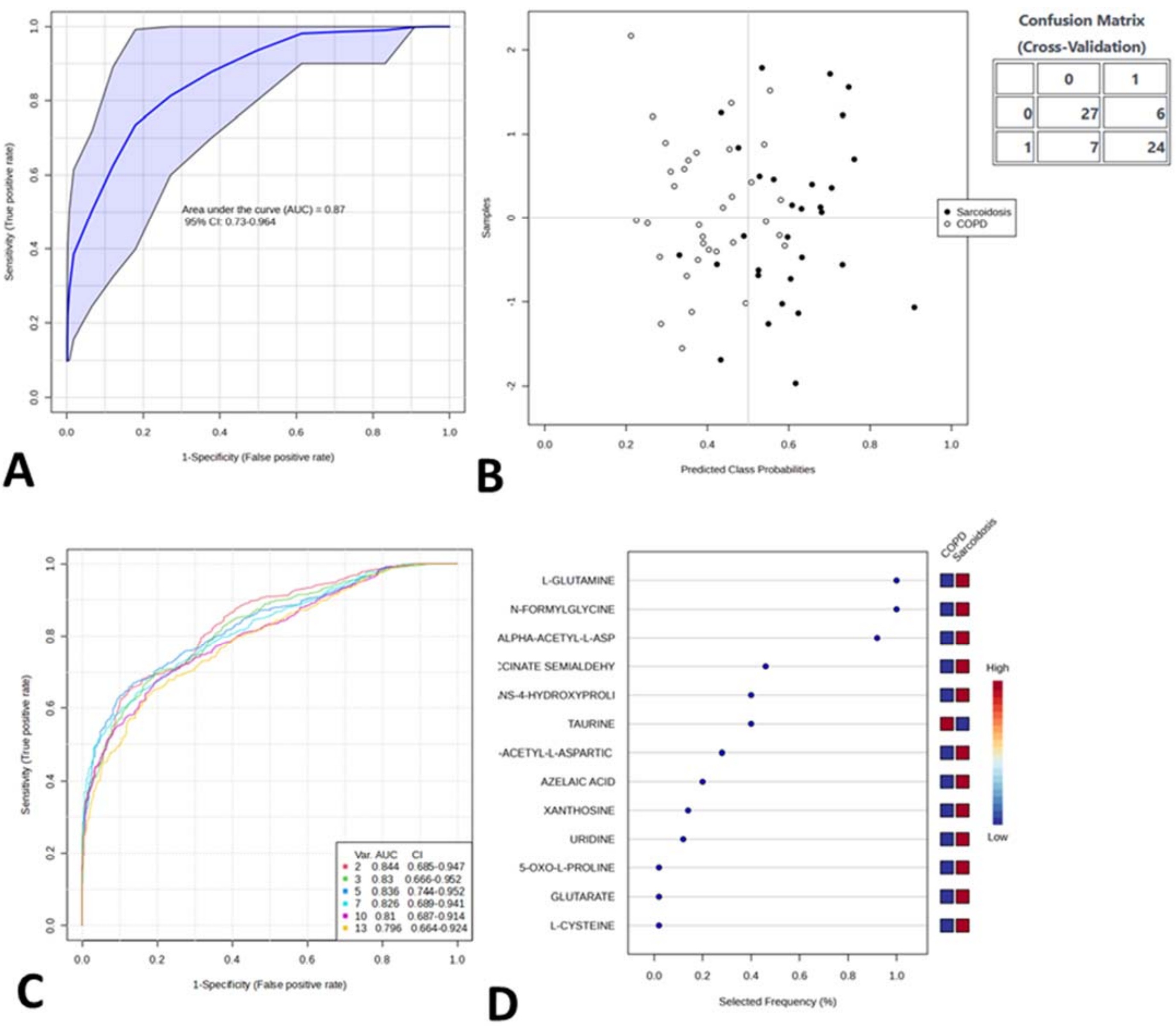

Figure S9. A: ROC based on the 6 metabolites to identify Sarcoidosis from COPD, B: The predicted class probabilities (average of the cross-validation) for each sample using the best classifier (based on AUC). The confusion matrix is also provided below the classifier image. C: ROC based on the different combinations of different metabolites. D: Significant metabolites and their importance in identifying Sarcoidosis from COPD.

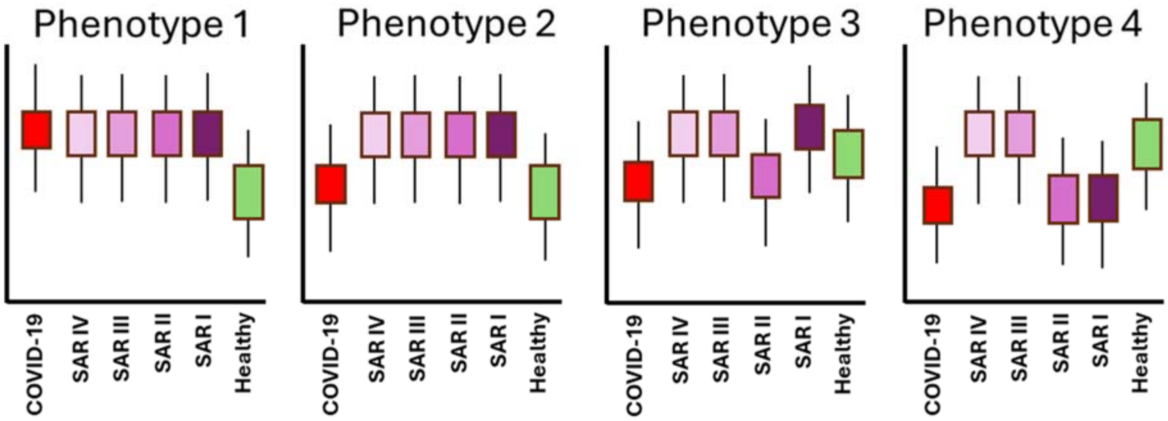

Figure S10. Schematic of the four primary metabolic phenotypes highlighting the distinct metabolic profiles across different stages of sarcoidosis, COVID-19, and healthy controls.

Table S2. The 4 main phenotypes are possibly linked to immune activation. Each phenotype is characterized by changes in among COVID-19, 4 stages of sarcoidosis and healthy controls through symbols (=): equal concentrations, (>), Increased concentrations and (<): decreased concentrations across the different groups.

Phenotype	Possibly linked to immune activation
COVID-19 = Sar IV = Sar III = Sar II = Sar I > Healthy Controls	**1a**: Elevated inflammatory metabolites in sarcoidosis stages 1, 3, and 4. Associated with acute inflammation and multi-organ involvement. **1b**: Shared inflammatory pathways in sarcoidosis stages 1 and 3, not elevated in stages 2 and 4. **1c**: Distinct inflammation profile in sarcoidosis stage 1 compared to other stages, highlighting disruptions in L-glutamine, ascorbate, and tryptophan metabolism.
COVID-19 < Sar IV = Sar III = Sar II = Sar I > Healthy Controls	Elevated metabolite levels across all sarcoidosis stages. Reflects chronic inflammation in sarcoidosis, with reduced levels in COVID-19 and healthy controls.
COVID-19 < Sar IV = Sar III > Sar II < Sar I > Healthy Controls	Elevated metabolites in sarcoidosis stages 1, 3, and 4, with reductions in stage 2 and in COVID-19 patients. Indicates stage-specific metabolic perturbations and chronic inflammation.
COVID-19 < Sar IV = Sar III > Sar II < Sar I < Healthy Controls	Elevated metabolites in healthy controls, moderate increases in sarcoidosis stages 3 and 4, and reductions in stages 1 and 2. Suggests overlapping profiles between chronic inflammation in sarcoidosis and baseline healthy states, contrasting with COVID-19's acute inflammatory response.
